# Postmortem Diagnosis of Dilated Cardiomyopathy: A Systematic Review Revisiting Fundamentals

**DOI:** 10.3390/diagnostics15233063

**Published:** 2025-12-01

**Authors:** Simona Calabrese, Vincenzo Cianci, Daniela Sapienza, Alessandro Nicolosi, Beatrice Spadaro, Antonio Ieni, Desirèe Speranza, Patrizia Gualniera, Alessio Asmundo, Cristina Mondello

**Affiliations:** 1 Department of Biomedical and Dental Sciences and Morphofunctional Imaging, Section of Legal Medicine, University of Messina, via Consolare Valeria, 1, 98125 Messina, Italy; simona.calabrese1@studenti.unime.it (S.C.); daniela.sapienza@unime.it (D.S.); alessandro.nicolosi@studenti.unime.it (A.N.); beatrice.spadaro@studenti.unime.it (B.S.); patrizia.gualniera@unime.it (P.G.); alessio.asmundo@unime.it (A.A.); 2 Department of Human Pathology in Adult and Developmental Age “Gaetano Barresi”, Section of Pathology, University of Messina, 98125 Messina, Italy; antonio.ieni@unime.it; 3 Department of Chemical, Biological, Pharmaceutical and Environmental Sciences, University of Messina, 98125 Messina, Italy; desiree.speranza@gmail.com

**Keywords:** dilated cardiomyopathy, DCM, postmortem analysis, postmortem genetic test, cardiomyopathy, sudden cardiac death

## Abstract

**Background:** Dilated cardiomyopathy (DCM) is a myocardial disorder characterized by structural and functional abnormalities, in particular left or biventricular chamber dilatation and systolic dysfunction, occurring without evidence of coronary artery disease, hypertension, valvular disease, or congenital heart defects. It is a significant cause of sudden cardiac death, particularly in young individuals, often remaining undiagnosed until autopsy. **Methods:** A systematic review of the literature was conducted following PRISMA guidelines to revisit the main postmortem findings (gross, microscopic, and genetic) useful to perform the postmortem diagnosis of DCM. Scientific databases (PubMed and Scopus) were searched for articles published up to February 2025 describing postmortem findings in individuals diagnosed with DCM. Inclusion criteria were focused on studies reporting macroscopic cardiac findings, and microscopic and genetic variants identified postmortem or in related familial studies. Data were extracted and categorized to identify consistent diagnostic markers and to assess the frequency and relevance of genetic findings in autopsy-confirmed DCM cases. From 2081 initial records, 30 studies met inclusion criteria. Two reviewers independently performed study selection and data extraction, and methodological limitations of the included studies were considered qualitatively to inform the synthesis. **Results:** Common macroscopic features included increased heart weight (often > 350 g), dilated left or biventricular chambers, and thinning of the ventricular walls. Histologically, the most consistent findings were diffuse interstitial fibrosis, myocyte hypertrophy, and nuclear atypia. Particular attention was given to morphological features essential to distinguish between genetic and nongenetic forms of DCM and, thus, useful to perform a differential diagnosis with disease having a DCM-like pattern. Notably, truncating variants in genes such as TTN, FLNC, DSP, PKP2, and MYH7 were frequently reported, particularly in young decedents with no significant history of cardiac disease. However, only about half of reviewed studies included any form of genetic analysis, reflecting a significant gap in current practice for forensic pathologists. **Conclusions:** DCM may cause sudden death without prior symptoms, making genetic testing essential to uncover the diagnosis, especially in cases with a negative phenotype. Therefore, molecular autopsy combined with careful macroscopic and microscopic analysis can strengthen the forensic assessment.

## 1. Introduction

Cardiomyopathies are a heterogeneous group of myocardial disorders characterized by mechanical and/or electrical dysfunction. These conditions are often—but not always—accompanied by abnormal ventricular hypertrophy or dilatation and can result from various causes, many of which are genetic in origin. They may be confined to the heart or occur as part of systemic diseases, frequently leading to cardiovascular mortality or progressive heart failure with associated disability [1]. These disorders are categorized into two main groups based on the extent of organ involvement. Primary cardiomyopathies (genetic, nongenetic, and acquired) are predominantly or exclusively limited to the heart muscle and constitute a relatively small subset. In contrast, secondary cardiomyopathies involve pathological myocardial changes as part of a wide range of systemic (multiorgan) diseases. Among the different types, dilated cardiomyopathy (DCM) can be classified as a mixed (genetic and nongenetic) nonischemic heart muscle disorder and is characterized by structural and functional myocardial abnormalities, in particular left or biventricular chamber dilatation and systolic dysfunction, occurring without evidence of coronary artery disease, hypertension, valvular disease, or congenital heart defects [1,2].

DCM is a leading contributor to heart failure requiring transplantation worldwide. It affects approximately 40 individuals per 100,000 in the general population, with an estimated annual incidence of 7 cases per 100,000. While notable ethnic disparities have been identified, sex-based differences appear to be less consistent. In the pediatric population, DCM accounts for nearly 60% of all cardiomyopathies, with the highest incidence observed in infants under one year of age [3].

A recent Mendelian randomization study has identified obesity and smoking as factors that are causally linked to an increased risk of developing dilated cardiomyopathy (DCM). In particular, genetically predicted elevations in body mass index (BMI) were strongly associated with a higher likelihood of DCM, with an odds ratio of 1.62 (95% confidence interval: 1.30–2.02; *p* = 1.51 × 10^−5^). These findings support a causal relationship between these modifiable risk factors and the development of nongenetic DCM [4].

Mutations in sarcomeric genes are frequently associated with DCM, often characterized by variable expressivity and incomplete penetrance. Recent advances in genetic research have demonstrated that the use of comprehensive gene panels significantly improves mutation detection, as clinical features alone are often insufficient to distinguish between different genetic subtypes. Among the most commonly implicated genes in DCM is TTN (titin), with truncating variants representing the most prevalent genetic cause. MYH7, which encodes the β-myosin heavy chain, is also frequently mutated in affected individuals. TNNT2, responsible for coding troponin T, is another gene in which pathogenic variants are often identified. TPM1, encoding α-tropomyosin, is similarly associated with recurring mutations in DCM. Although MYBPC3 mutations are well known in other cardiomyopathies, they appear to be less common in DCM. In addition to sarcomeric genes, mutations in LMNA (lamin A/C), SCN5A, and OBSCN (obscurin) have also been observed, highlighting the considerable genetic and mechanistic heterogeneity underlying this condition [3,5].

The aim of this review is to systematically analyze the postmortem/forensic literature describing DCM features to provide insight on macroscopic, microscopic, and genetic findings, which can allow for effective postmortem diagnosis. Then, specific histological data are discussed to focus on the main evidence that can help pathologists in defining the differential diagnosis of DCM.

## 2. Materials and Methods

This systematic review was conducted in accordance with the 2020 PRISMA guidelines (Supplementary Material File S1) [6], utilizing the PubMed and Scopus databases between 8 January 2025 and 28 February 2025. The main search lines are as follows. In particular, the thorough search of PubMed made use of the following: (“Dilated cardiomyopathy” OR “DCM”) AND (forensic OR postmortem) AND (“gross examination” OR “histopathology” OR “imaging” OR “postmortem imaging” OR “genetics” OR forensic genetics). In Scopus, a search was performed using the following string: TITLE-ABS-KEY ((“dilated cardiomyopathy” OR “DCM”) AND (forensic OR postmortem) AND (“gross examination” OR “histopathology” OR “imaging” OR “postmortem imaging” OR “genetics” OR “forensic genetics”)).

The complete and fully reproducible search strategies for each database, reported exactly as entered in the search platforms and without any modifications or filters, have been provided. Secondary searches included the screening of reference lists of the included articles that met the inclusion criteria. The primary criterion for article selection was the presence of terms or concepts in the title and/or abstract indicating the forensic or postmortem analysis of DCM cases. If the abstract suggested potential relevance, the full article was reviewed to assess its eligibility. Studies were excluded at the title, abstract, or full-text level if they did not address the topic of interest. Additionally, review articles, editorials, letters to the editor, and studies on DCM associated with genetic syndromes were excluded. The selected articles underwent an in-depth analysis, focusing on macroscopic and histological findings and genetic data. The systematic review was carried out in accordance with the following standards to lower the possibility of bias. Two writers (S.C. and C.M.) independently completed the initial stage of the article selection procedure, which was restricted to the title and/or abstract. The articles that seemed to fit the requirements for inclusion were completely read. Lastly, the review included papers that were deemed to meet the inclusion criteria by three authors (A.A., D.S., and V.C.). The degree of agreement between the two researchers was then assessed using Cohen’s Kappa test, which showed a high degree of concordance (κ > 0.8). Three investigators (C.M., V.C., and P.G.) extracted the data, and two more authors (A.N. and B.S.) confirmed the results. Discrepancies were resolved by consensus; unresolved conflicts were adjudicated by a third reviewer. Gray literature was not included, as our search strategy focused on peer-reviewed postmortem studies to ensure consistency in the diagnostic information reported.

**Figure 1:**
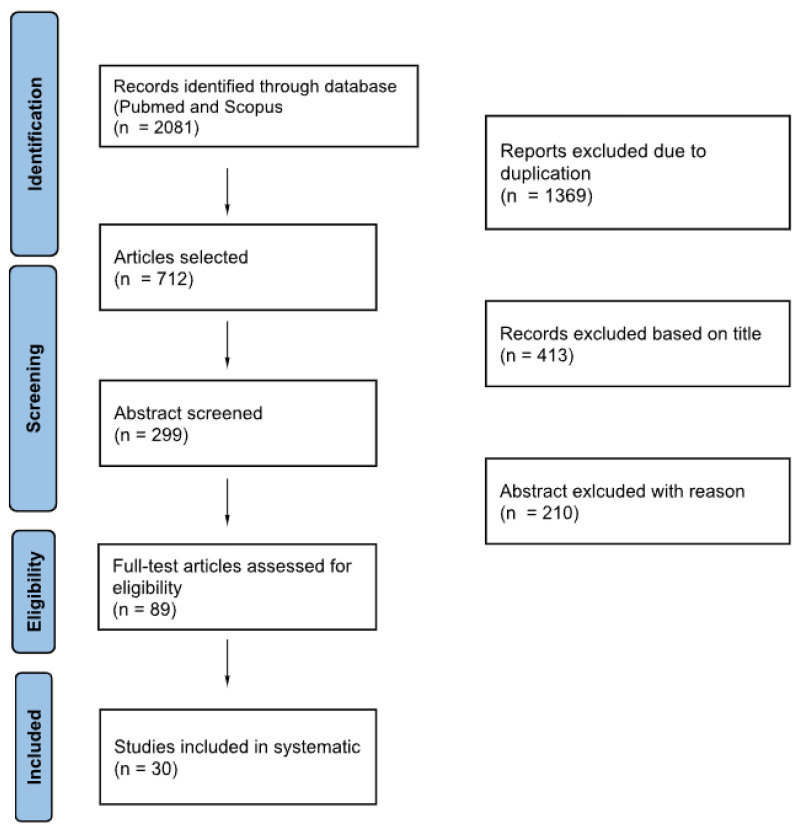
Flowchart of literature selection process.

A flowchart is reported in Figure 1, following the PRISMA guidelines.

### 2.1. Data Synthesis and Management of Heterogeneity

A meta-analysis was not possible due to the heterogeneity of the included studies, which were primarily case reports and small case series with varying population characteristics, pathological reporting standards, and levels of genetic evaluation. As a result, a narrative synthesis method was used. Heterogeneity was managed by grouping findings into three predefined domains (macroscopic, microscopic, and genetic features) and summarizing recurring patterns within each domain. Qualitative comparisons between macroscopic and microscopic results were made, with an emphasis on components that were consistently reported in various studies. Sarcomeric, desmosomal, cytoskeletal, and mitochondrial gene families were used to classify genetic variants, which were then combined based on their potential pathogenicity and recurrence. This structured thematic synthesis allowed for the integration of highly heterogeneous data while maintaining comparability across studies. Genetic variants were extracted as reported by the original studies and grouped by gene function. Variant interpretation followed the classifications provided by the authors (generally based on ACMG/AMP criteria). In cases of conflicting classifications, we referred to the designation used in the autopsy report or the most recent source.

### 2.2. Certainty of Evidence

The conceptual domains of the GRADE framework were considered to evaluate the certainty of the evidence. However, because the available literature consists almost entirely of single case reports and small case series, study designs for which a formal GRADE rating is not methodologically applicable, a quantitative GRADE table was not produced. Although heterogeneity in autopsy procedures, sampling strategies, and reporting standards limits the precision of conclusions, the recurrence of consistent macroscopic, microscopic, and genetic patterns across studies supports a moderate level of conceptual certainty at the descriptive–pathological level.

Similarly, validated risk of bias tools (e.g., JBI, NOS, and ROBINS-I) cannot be meaningfully applied to isolated case reports or very small autopsy series. In line with recommendations for descriptive pathological evidence syntheses, we therefore adopted a structured qualitative assessment, evaluating all studies across a predefined set of domains: reporting completeness, sampling clarity, macroscopic–microscopic concordance, and transparency of analytical techniques. This narrative but systematic framework ensured a reproducible appraisal without generating artificial or non-interpretable scores.

### 2.3. Eligibility Criteria

Original postmortem studies that reported macroscopic, microscopic, and/or genetic findings consistent with a dilated cardiomyopathy (DCM) diagnosis were included. Since case reports and small case series are the most common source of autopsy-based evidence for DCM-related sudden death, they were deemed eligible due to the nature of the forensic literature. Studies had to offer enough pathological information to support the diagnosis, including at least one of the following: identification of pathogenic/likely pathogenic variants linked to cardiomyopathies, characteristic microscopic features (such as myocyte hypertrophy and interstitial or replacement fibrosis), or ventricular dilatation at gross examination. Review papers, editorials, conference abstracts, and studies without postmortem pathological information were not included. Additionally, because their complex systemic involvement may confuse the isolated cardiac phenotype relevant to nonsyndromic DCM, we excluded cases of cardiomyopathy occurring as part of syndromic or multisystem genetic disorders, such as muscular dystrophies or metabolic storage diseases. This method made it possible to concentrate particularly on autopsy results linked to primary, nonsyndromic DCM.

### 2.4. Limitations and Risk of Bias

The majority of the included studies are case reports and small case series, which is a significant limitation of this review. The descriptions of macroscopic, microscopic, and genetic findings are characterized by variability and inherent limitations on generalizability. Consequently, conclusions should be interpreted cautiously because the overall strength of the evidence is still low. No review protocol was prospectively registered. The review was conducted following predefined methodological criteria chosen by the authors, but the absence of a registered protocol may have increased the risk of bias. Heterogeneous reporting and the predominance of single case or very small case series reports limited the overall quality of the included studies, which weakens the evidence and should be taken into account when interpreting our findings. Additionally, the evidence base was extremely diverse and mostly consisted of low-tier, descriptive designs (single case and small case series reports). Comparability was limited by inconsistent application of histological criteria across studies, and the integration of morphological and molecular findings was hampered by incomplete genetic data. Due to the heterogeneity of the included studies (experimental models, proteomic analyses, histochemistry, case reports, and small autopsy series), a formal risk of bias assessment using clinical tools (e.g., JBI) was not considered methodologically appropriate. Instead, study quality was appraised narratively by considering the clarity of sampling, reporting of PMI and environmental conditions, and transparency of analytical methods.

## 3. Results

The main data of the enrolled articles are reported in Table 1, Table 2 and Table 3.

### 3.1. Macroscopic Findings

The main gross findings obtained from the reviewed cases reveal a wide range of cardiac changes, primarily involving chamber dilation and significant alterations to myocardial architecture. A comparative analysis of autopsy findings shows recurring and distinctive elements, such as cardiomegaly, biventricular dilation, and variable wall thicknesses, all of which reflect the pathophysiological progression of the disease [7,8,9,10,11,12,13,14,15,16,17,18,19,20,21].

Heart weight, a sensitive marker of cardiac hypertrophy and remodeling, was reported in most of the studies, with values ranging broadly according to patient age and DCM subtype. Adult cases commonly exhibited heart weights between 350 and 900 g [7,8], markedly exceeding the upper limit of normal for both sexes. In pediatric cases, values adjusted to age and body size still reflected pathological hypertrophy, for instance, 77–250 g in children aged 2–11 years [9] and as low as 22.4–39.7 g in neonates [10], all indicating disproportionate cardiac mass relative to body surface area.

In terms of chamber wall thicknesses, several studies detailed both absolute values and qualitative descriptions. Benjamin et al. reported LV wall thicknesses ranging from 0.7 to 1.65 cm, suggesting a trend toward eccentric hypertrophy with wall thinning in late-stage disease [7]. Okamoto et al. (1993) described a septum of 18 mm, an LV posterior wall of 13 mm, and an RV wall of 5 mm, which, although increased in absolute terms, must be interpreted in the context of concurrent chamber dilation—consistent with eccentric remodeling rather than concentric hypertrophy [11]. Chen & Zhang (2006) noted LV thicknesses from 0.8 to 1.9 cm and interventricular septum thicknesses from 1.0 to 1.4 cm, further supporting this model of disproportionate expansion with preserved or modestly thickened walls [12].

Across the dataset, cardiomegaly was universally documented, appearing in every case where the heart was grossly described [8,9,12,20,21]. Additionally, biventricular dilatation appeared as a hallmark finding in several forms of DCM [7,11,15,19,21], confirming the disease’s systemic myocardial involvement, except in the rare case described by Samanta et al. [13], in which an isolated dilation of the right atrium and ventricle was reported, and in the case of Zhang et al. [17], where right ventricular dilation was found in 7 out of 11 cases of cardiomyopathy in obstructive sleep apnea. Isolated left ventricular dilatation, as noted by Matoba et al. [14] and Takahashi et al. [16], was rarer and possibly representative of early-stage or age-related anatomical response in pediatric or acute-onset cases.

In some cases, additional features such as endocardial thickening and whitish fibrotic plaques were reported, suggestive of chronic wall stress and fibrosis [16,18]. The endocardial fibrosis was usually focal and, in several cases, it was observed at the apex. Moreover, the apex was described as an endocardial site in which were frequently observed thrombi in various stages of organization [8]; these endocardial thrombi were described in small recesses within trabecular myocardium.

### 3.2. Microscopic Findings

The histopathological examination across the reviewed cases of dilated cardiomyopathy revealed a consistent pattern of structural myocardial abnormalities, although with some heterogeneity reflecting etiological and age-related variability [7,8,9,10,11,12,13,14,15,16,17,18,19,20,21,22,23]. In the study by Benjamin et al. [7], focal areas of myocardial fibrosis were identified, suggestive of chronic myocyte injury and remodeling [7]. Matsubara et al. [8] reported disorganization of myocardial fibers in the left ventricle in two of seven cases, accompanied by focal mononuclear cell infiltrates and increased interstitial fibrous tissue, findings that may reflect both degenerative and inflammatory processes. In pediatric cases associated with AIDS, Joshi et al. [9] observed myocardial fiber hypertrophy in three out of five children, along with interstitial edema and infiltration by mononuclear inflammatory cells, highlighting a possible immunopathogenic component. Similarly, Matoba et al. [14] described diffuse interstitial fibrosis particularly localized in subendocardial and perivascular areas, reinforcing the role of chronic ischemia and pressure overload. A more recent and detailed analysis by Pelletti et al. in 2021 [21] described diffuse non-specific alterations, including mild subendocardial fibrosis, also described by Fernlund et al. [23], and mild to moderate interstitial myocardial fibrosis, which had a nonischemic distribution and was more noticeable in the lateral left ventricular wall and septum. This study also noted an increase in cardiomyocyte size with irregularly shaped nuclei and mild cytoplasmic vacuolization, while confirming the absence of recent ischemic lesions, inflammation, or signs of toxic myocardial damage [21]. A case reported by Simoes et al. [15] demonstrated a diffuse pattern of myocyte hypertrophy with patchy interstitial fibrosis, supporting the diagnosis of idiopathic DCM with typical remodeling features. Hypertrophic cardiomyocytes were also found by Callon et al. [22], associated with interstitial and scarring fibrosis [22]. It must be highlighted that microscopic interpretation may be affected by postmortem autolysis, which can obscure cellular details and reduce the reliability of specific histological features in some cases.

### 3.3. Genetic Analyses

Genetic analysis of the reviewed DCM cases revealed a highly heterogeneous mutational landscape, involving both nuclear and mitochondrial genomes [23,24,25,26,27,28,29,30,31,32,33,34,35,36]. Among the most extensively represented alterations, Ruppert et al. [24] identified a significant burden of mitochondrial DNA (mtDNA) mutations in patients with non-familial DCM, particularly in genes encoding NADH dehydrogenase and cytochrome c oxidase subunits. Notable variants included c.4079A>G, c.4588C>T, and D-loop mutations such as c.16189T>C, alongside tRNA mutations and novel missense changes (e.g., c.5973G>A and c.7042T>G) affecting conserved protein domains, supporting a pathogenic role in myocardial bioenergetics.

On the nuclear genome side, Elliot et al. [25] reported a range of missense and splicing mutations in desmosomal genes, including PKP2 (S140F and H877Q) and DSP (IVS15–1G>C and A2712T), alongside variants of uncertain significance such as V56M (DSG2) and G863R (DSC2), typically found in cases of arrhythmogenic right ventricular cardiomyopathy reported in literature (ARVC).

Mutations in sarcomeric genes have also been documented. Murakami et al. [26] identified a TNNI3 Pro16Thr missense variant in a sporadic DCM case, suggesting the involvement of thin filament protein dysfunction even in non-familial forms. Additionally, Fernlund et al. [23] highlighted the co-occurrence of TNNT2 and BAG3 variants in familial DCM with early-onset and malignant progression, pointing to potential gene–gene interactions influencing disease expressivity. Finally, Kraoua et al. [34] reported a frameshift mutation in TNNI3 within a consanguineous family, linked to fatal infantile dilated cardiomyopathy. Dong et al. [32] described three novel TTN mutations, including a splice-site variant (c.35485+1G>A) and two frameshift mutations (c.82137del and c.80415insA), all located within functionally critical domains of titin. These variants further substantiate TTN as one of the most frequently mutated genes in DCM. Other relevant findings include a MYBPC3 synonymous mutation (c.24A>C and p.P8P) in the case of sudden cardiac death regarding a 29-year-old woman diagnosed with DCM [33], a novel CASZ1 frameshift mutation with multisystem involvement [30], and a nonsense mutation in TXNRD2 along with a duplication in FLNC, both reported by Rojnueangnit et al. [29].

Single nucleotide polymorphisms (SNPs) were explored by Dou et al. [27] in the NAMPT gene, where specific haplotypes (e.g., T-A-C) were associated with protective effects against DCM, while rs9034 variants correlated with prognosis and survival, positioning this locus as a candidate prognostic biomarker.

Structural variants such as copy number variations (CNVs) were examined by Mates et al. [28], who found a 4.4% prevalence in DCM patients, significantly higher than in previous cohorts, reinforcing the need to integrate CNV screening into genetic testing protocols. Similarly, truncating variants in FLNC (filamin C), as reported by Hespe et al. [31], have been strongly associated with arrhythmogenic phenotypes overlapping with DCM, often accompanied by myocardial fibrosis and systolic dysfunction.

Furthermore, Saxton et al. [35] and Lorca et al. [36] emphasize the recurrent involvement of RBM20, MYH7, SCN5A, and PKP2 in DCM, particularly the RBM20 p.Arg636Cys variant, which has been linked to a high risk of sudden cardiac death even in subclinical presentations.

## 4. Discussion

The studies included in this review consistently confirmed that postmortem diagnosis of DCM relies primarily on structural evidence rather than clinical phenotypes. Across the literature, phenotypic variability was reflected in the heterogeneity of macroscopic and microscopic patterns, which converged nonetheless into a limited set of recurring autopsy hallmarks [37,38,39]. In this context, we clarified the distinction between clinical diagnostic criteria for DCM, such as systolic dysfunction, LVEF-based classifications, and ESC-defined phenotypes, which cannot be directly assessed postmortem, and the structural macroscopic and microscopic features that form the basis of the autopsy diagnosis, including chamber dilatation, heart weight, myocardial fibrosis, and myocyte hypertrophy.

In our synthesis, heterogeneity in etiology mainly influenced the variability in histological findings, particularly the amount and distribution of fibrosis. However, the overall macroscopic profile remained remarkably consistent across different causes of DCM, supporting its diagnostic value in autopsy settings. Such an integrated framework is clinically relevant, as specific etiologies and genotypes are associated with distinct arrhythmogenic profiles, progression rates, and therapeutic implications. Incorporating both structural features and causal mechanisms could therefore provide a comprehensive actionable approach to DCM classification [40].

When considering the macroscopic findings together, the reviewed studies revealed a reproducible pattern: increased heart weight, dilation of one or both ventricles, and disproportionate eccentric remodeling. These converging features allow pathologists to distinguish DCM from differential diagnoses such as HCM or ischemic cardiomyopathy, where remodeling follows different geometric patterns [7,8,9,13,20,21].

The coexistence of chamber dilation and variable myocardial thickening also illustrates the complex adaptive mechanisms of the failing heart: initially compensating through hypertrophy, and eventually decompensating into dilatation and systolic failure [7,11,15,19,21]. These macroscopic changes are not merely descriptive but carry prognostic significance, explaining its development into disease chronicity and hemodynamic burden. In fact, as already stated by Bortone et al., chamber properties rely on the thickness and volume of the left ventricle, whereas myocardial integrity reflects the molecular pattern of the myocardial tissue [41]. As reported in the literature, when the left ventricle is not capable of fulfilling the body’s demands, heart failure occurs [42].

The macroscopic findings appear to be often correlated with our microscopic data showing interstitial and replacement fibrosis with a nonischemic distribution, and are relevant for understanding the arrhythmogenic substrate of advanced DCM [8,14,15,18,19]. Indeed, increased amounts of interstitial fibrous tissue have been found to be associated with increased myocardial stiffness [41]. Myocyte hypertrophy, according to the gross appearance of hypertrophy, is also a frequent finding, consisting of a marked increase in cell area with enlarged hyperchromatic and asymmetric nuclei [15,21,23]. Collectively, these findings underscore the central role of myocyte hypertrophy and interstitial fibrosis as histopathological hallmarks of DCM, regardless of the underlying etiology. Myocytes can also show hydropic changes due to myofibril loss, ranging from perinuclear halo to colliquative myocitolysis [21]. Such alterations reflect progressive myocardial degeneration, compensatory remodeling, and contractility failure [43,44]. Signs of low-grade interstitial inflammation were also observed, like focal sparse lympho-monocyte infiltrates, mainly close to areas of replacement fibrosis [8,9,45].

The gross and microscopic evidence showed no specific findings or degree of fibrosis or myocyte modification; thus, forensic pathologists may have difficulties in performing differential diagnosis between genetic and nongenetic (acquired) DCM. The main diseases showing a DCM-like pattern include myocarditis, end-stage hypertrophic cardiomyopathy (HCM), arrhythmogenic cardiomyopathy (ACM), chronic myocardial ischemia, and isolated heart sarcoidosis [45]. Clinical history and/or histological findings can help pathologists in differential diagnoses.

The phenotype of end-stage HCM can be characterized by ventricular dilatation mimicking DCM [46,47,48]. This dilatation occurs in the advanced phase of HCM due to the marked and broad fibrotic replacement of myocardial tissue. As a result, the macroscopic and microscopic areas of fibrosis may be more pronounced compared to those of DCM and, microscopically, can coexist with areas of myocyte disarray [11,49]. Moreover, in end-stage HCM, the dilatation can coexist with the hypertrophic sites of left ventricle walls [47,49]. The narrowing of intramyocardial arteries, mainly characterized by the increased thickness of the wall due to hypertrophy of the media and intimal, can be another finding of end-stage HCM not observable in DCM [47,50,51].

A fibrotic pattern determining wall thinning and chamber dilatation can be observed in chronic ischemic heart disease due to multiple ischemic events resolving with fibrotic substitution of myocardial tissue; however, in these cases, the distribution of fibrosis is mainly spread across the myocardial areas dependent on the vessel obstructed by atherosclerosis [52,53].

The differential diagnosis of DCM also includes ACM, mainly in biventricular- and left ventricular-dominant ACM variants [45]. The first phenotype frequently showed both replacement fibrosis starting from the epicardium or mid-mural layers and a fibro-fatty pattern in the right ventricle; in the second one, the left ventricle mainly showed interstitial and replacement fibrosis with little fat, making the differential diagnosis harder [54]. Potential findings which may support the diagnosis of ACM rather than DCM are related to the quantification of the percentage of fibrous and/or fibro-adipose tissue replacement. Some reports in the literature on ACM-related sudden cardiac death identify a ventricular wall infiltration of at least 3% adipose tissue and over 40% fibrous tissue (in seven microscopic fields at 400× magnification) [55].

DCM can also result from the progression of myocarditis (so-called inflammatory DCM), especially in subacute and chronic forms [56,57]. Histologically, these cases can show fibrosis at several stages (interstitial/replacement fibrosis, subendocardial fibrosis, and endocardial fibrosis), myocyte damage/necrosis, and interstitial and/or endocardial inflammation [58]. The use of immunohistochemistry to characterize inflammatory cell immunophenotypes may help in making the differential diagnosis [59]. The main markers are CD45 (leukocyte antigen common), CD43 (T lymphocytes), CD3 (T cell marker), CD4 (helper T cell marker), CD8 (cytotoxic T cell marker), CD45RO (memory subset of CD8+ T cell marker, expressed on activated T cells), CD20 (B-lymphocytes), and CD68 (macrophages) [59]. Moreover, in cases with gross findings of DCM, it is suggested that an infiltrate of ≥14 lymphocytes and macrophages/mm2 (diffuse, focal, or confluent), quantitated by immunohistochemistry on an endomyocardial biopsy sample, can be considered a cut-off to reach the diagnosis of inflammatory DCM [60].

Viral infections are the leading infectious causes of myocarditis and inflammatory dilated cardiomyopathy, and many studies have confirmed the presence of viral genomes in myocardial tissue using PCR-based methods. Systematic evidence shows that enteroviruses and parvovirus B19 are among the most frequently detected pathogens, with positivity rates often reaching one-third or more of biopsy samples, while EBV, adenovirus, HHV-6, and CMV appear less consistently. Similar findings are reported in children, where enteroviruses and B19 are also prominent and may be identified in over 40% of cases, with occasional involvement of other viruses such as HHV-7. Overall, the literature supports that viral markers are common in both adult and pediatric myocarditis and DCM, underscoring the importance of molecular viral testing in clinical evaluation [61].

A DCM-like macroscopic phenotype can be also observed in sarcoidosis [62]. However, sarcoidosis offers many different microscopic findings, represented by “true granulomas” (non-necrotizing, small, compact, and epithelioid, frequently found in the free walls of the left ventricle and in the interventricular septum) composed mainly by macrophages and multinucleated giant cells, and fibrosis with foci of lymphocytes or lymphohistiocytes outside the granuloma [63].

Notably, current evidence indicates that DCM may involve multiple organ systems rather than being confined to the myocardium. Alterations in thyroid hormone metabolism have been documented frequently in patients with DCM, and both clinical observations and experimental models show that thyroid dysfunction can adversely influence patient outcomes [64].

Extracardiac pathology has also been observed in the lungs. In a large autopsy series by Soeiro and colleagues involving 186 individuals with DCM who died from acute respiratory failure, histological examination frequently revealed diffuse alveolar damage, pulmonary edema, and alveolar hemorrhage [65].

These findings highlight the value of comprehensive autopsy studies, particularly when the clinical phenotype is unclear, to support the diagnosis of DCM and characterize its systemic involvement.

Within the broad spectrum of DCM, the genetic test has a relevant role in performing postmortem diagnosis [66,67,68]. Pathogenic gene variants can frequently be a direct cause of the disease, and can implicate many genes and several pathways. Moreover, several reports in the literature have highlighted the interplay between genetic and acquired causes (i.e., hypertension, toxins, and excessive use of alcohol), stressing that the presence of an acquired condition does not exclude the simultaneous occurrence of a causative gene variant [69].

**Figure 2:**
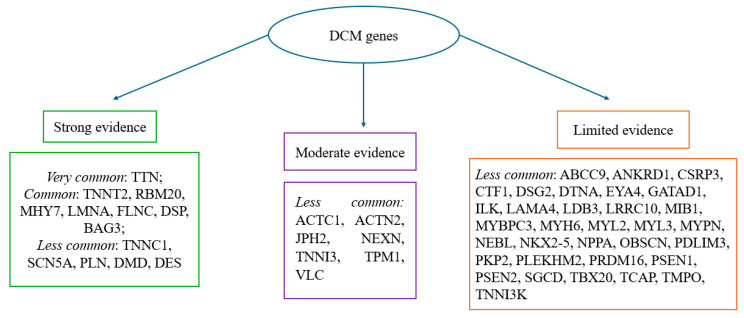
Frequency and strength of evidence of genes associated with DCM; most common: >10% of cases; common: 1–10% of cases; less common < 1% of cases. The categories reported in Figure 2 (“frequency and strength of evidence of genes associated with DCM”) reflect the number of included studies in which each gene was identified, in accordance with the ESC 2023 guidelines [39].

Moreover, recent studies have demonstrated that the phenotypic expression of the disease could not be observed at postmortem heart examination, despite the underlying mechanism of death having DCM genetic patterns. Several studies reported that genetic variants may be present even in the absence of overt structural abnormalities, a finding that is particularly relevant for unexplained sudden deaths in the young. This emphasizes the need to correlate subtle histological patterns with molecular results, especially when the macroscopic phenotype is equivocal. These phenotypes are strongly related to the expression of certain gene mutations (such as LMNA for conduction defects). Specifically, the latter phenotype clinically describes global systolic impairment of the left or both ventricles (LVEF < 45%) without chamber dilatation, and not due to abnormal loading conditions or coronary artery disease. In many individuals—for example, relatives who are mutation carriers or exhibit anti-heart antibodies—there is a preclinical phase without cardiac expression that subsequently progresses towards mild cardiac abnormalities. The overt phase of systolic dysfunction is usually associated with LV dilatation, but this may be absent in some cases, causing diagnostic confusion [67]. Thus, understanding the underlying mechanisms of DCM is crucial, since it can stem from various genetic mutations, which can be divided into two broad categories: problems in force generation and impairments in force transmission. Force generation defects usually result from abnormalities within the sarcomere, the core contractile structure of heart muscle cells. Force transmission impairments are caused by mutations in genes, such as those encoding for dystrophin and desmin, which play a critical role in relaying contractile force from the sarcomere to the cell membrane and surrounding tissue [69]. The main genes involved in the onset of DCM are reported in Figure 2 and represent what is stated in the ESC 2023 guidelines regarding the most common mutations in DCM patients. The frequency thresholds were set according to systematic reviews from international evidence-based literature [39].

In our study, we found that the most frequently represented mutations in DCM span a wide array of genes involved in mitochondrial function (mtDNA), sarcomeric integrity (TTN, MYBPC3, and TNNT2), cytoskeletal architecture (FLNC and BAG3), and intercellular adhesion (DSP and PKP2), reflecting the complex and multifactorial nature of the disease [23,24,25,26,27,28,29,30,31,32,33,34,35,36]. The ESC guidelines identified the most common mutations involved in the pathogenesis of nonsyndromic cardiomyopathies [39]. Equally important is the use of radiological methods for a better characterization of postmortem diagnosis [70]. Our results appear to be linear with what is stated by the ESC, and outline how difficult it is to make a strict phenotypic delineation between arrhythmogenic and dilated cardiomyopathies. This is due to the genes involved often being common, as demonstrated by the desmosomal mutations (PKP2, DSP, DSG2, and DSC2) caused by genes typically associated with ARVC genes [25], FLNC truncating variants that lead to DCM with arrhythmogenic features [31], and RBM20 (p.Arg636Cys), MYH7, SCN5A, PKP2, and mutations predisposing individuals to arrhythmic risk and sudden cardiac death [35,36]. Interestingly, we found that the genetic overlap is not only limited to arrhythmogenic cardiomyopathies, but also extends to hypertrophic cardiomyopathy (HCM). Notably, sarcomeric genes such as TNNI3, TNNT2, MYBPC3, and MYH7 are classically associated with HCM, but the findings obtained by the present review demonstrate their significant role in DCM as well, establishing them as shared genetic substrates between the two cardiomyopathies [23,26,33,34,35,36].

The convergence of these mutations across studies underscores the necessity for comprehensive genetic screening, including analysis of SNVs, CNVs, and synonymous variants in both familial and sporadic cases of DCM, remembering that DCM is considered familial if one or more first- or second-degree relatives are affected by the disease, or when an otherwise unexplained SCD has occurred in a first-degree family member, regardless of the age, with a confirmed diagnosis of DCM [39]. Thus, especially in cases where the DCM phenotype is not typically represented, it appears necessary to integrate genetic findings with a detailed postmortem gross and microscopical examination, particularly in cases of unexplained sudden cardiac death in young individuals. The patterns emerging from our synthesis align with current autopsy recommendations, including the 2023 ESC cardiomyopathy guidelines and recent guidance for sudden unexplained death investigations—which highlight the need for structured cardiac sampling and integrated genetic assessment—but also the internationally recognized autopsy guidelines, including the Royal College of Pathologists’ guidelines on Autopsy Practice for sudden cardiac death, which underline the importance of structured cardiac dissection, systematic histological sampling, and careful clinicopathological correlation in the postmortem evaluation of suspected DCM [39,71].

### Implications for Forensic Practice and Future Directions

In light of the findings, some important implications for forensic practice emerge. A systematic approach to postmortem examination in suspected DCM cases should include accurate documentation of macroscopic findings (cardiac weight, chamber dilation, and any mural thrombi) and standardized histological sampling, with particular attention to patterns of interstitial and replacement fibrosis. When available, integrating postmortem genetic data provides useful diagnostic support and can also contribute to familial risk assessment. Looking ahead, forensic research would benefit from the adoption of more uniform protocols for sampling and histological evaluation, as well as from studies that more systematically integrate genetic analyses and new “omics” technologies, in order to improve the characterization of the relationships between the postmortem structural phenotype and molecular variants. Multicenter studies with shared diagnostic criteria could also facilitate greater comparability of findings and a more robust definition of the morphological patterns associated with DCM in cases of sudden death.

## 5. Conclusions

In conclusion, despite DCM often being identified through clinical assessment, the disease still represents one of the main causes of nonischemic SCD. While early recognition and management of DCM may improve outcomes, forensic pathologists can play a pivotal role in cases where the disorder is clinically silent, and where the diagnosis can only rely on autopsy findings and genetic testing. The literature reports the main genetic mutations associated with the disease and, despite the overlap with other forms of cardiomyopathies, these can guide the examiner towards a more precise forensic answer through the so-called molecular autopsy. Ongoing research on the main histopathological and molecular features of the various forms of DCM is necessary in order to enable a more precise and effective diagnosis, as current data are still limited. Integrating family screening and postmortem findings is crucial to achieve an early diagnosis and the prevention of SCD. Therefore, from a forensic perspective, these findings support the value of systematic genetic testing, standardized cardiac sampling, and careful documentation of macroscopic and microscopic features to improve the postmortem evaluation of suspected DCM.

## Figures and Tables

**Table 1:** Main macroscopic data of the enrolled articles. dnr: data not reported; RV: right ventricle; LV: left ventricle; LVWP: left ventricle posterior wall.

Authors	Year	Sample (n)	Heart Weight	DCM Type	Thicknesses	Gross Findings
Benjamin et al. [7]	1981	30 (mean 42.5 years)	350–900 g	Idiopathic	LV wall (0.7–1.65 cm)	Biventricular biatrial dilatation (all cases); ventricular mural thrombi (22 cases); normal myocardium (all cases)
Matsubara et al. [8]	1984	7 (25–66 years)	350–700 g	Congestive	dnr	Cardiomegaly (all cases);
Joshi et al. [9]	1988	5 (2–11 years)	77–250 g	AIDS-related	dnr	Cardiomegaly (all cases); biventricular dilatation (all cases); no mural thrombi (all cases)
Chang et al. [10]	2010	5 (22–67 days)	22.4–39.7 g	Mitogenic	dnr	Biventricular dilatation; cardiomegaly; endocardial fibroelastosis
Okamoto et al. [11]	1993	1 (15 years)	875 g	End-stage HCM	VS (18 mm); LVPW (13 mm); RV wall (5 mm)	Marked biventricular dilatation and hypertrophy
Chen & Zhang. [12]	2006	11 (14–49 years)	330–570 g	dnr	LV wall (0.8–1.9 cm); VS (1.0–1.4 cm)	Biventricular dilatation (all cases)
Samanta et al. [13]	2011	1 (37 years)	532 g	Idiopathic right ventricular	RV wall (4 mm); LV wall (11 mm)	RA and RV dilatation; multiple mural thrombi; no significant dilatation of the LV wall; left atrium slightly dilated; grade II atherosclerosis of aorta
Matoba et al. [14]	1990	1 (22 years)	420 g	Inflammatory DCM	dnr	Moderate dilatation of left ventricle and slightly dilated right ventricle; organized thrombus in the left atrium
Simoes et al. [15]	1992	1 (6 years)	350 g	Idiopathic	dnr	Mild thickening of the left ventricular endocardium; anteroapical transmural infarction in the healing stage; normal cardiac valves and no thrombi; free epicardial coronary arteries
Takahashi et al. [16]	2008	1 (4 months)	88 g	dnr	Right heart wall (0.5 cm); left heart wall (1.0 cm)	Left ventricular dilatation; whitish opacity on the endocardial surface
Zhang et al. [17]	2013	11 (19–71 years)	490–1000 g	dnr	RV wall (6.1 ± 1.6 mm); LV diameter > 4 cm (6 cases)	Right ventricular dilatation (7 cases)
Crauciuc et al. [18]	2021	62 (18–89 years)	dnr	Alcoholic	dnr	dnr
Ito et al. [19]	2021	5 (28–64 years)	410–730 g	dnr	LV wall (4–11 mm)	Severe dilatation of the bilateral ventricles

**Table 2:** Main microscopic data of the enrolled articles.

Authors	Year of Publication	DCM Type	Microscopic Findings
Benjamin et al. [7]	1981	Idiopathic	Small areas of focal fibrosis
Matsubara et al. [8]	1984	Congestive	Disorganized cells in the LV (2 cases); cardiac mural thrombi (4 cases); extensive interstitial fibrosis (2 cases); little stenosis of extramural coronary arteries (all cases); prominent intimal cushions in small arteries (all cases)
Joshi et al. [9]	1988	AIDS-related	Hypertrophy of myocardial fibers (3 cases); interstitial edema (all cases); mononuclear and lymphocytic inflammatory infiltrate (3 cases); focal intimal fibrosis or medial calcification of small branches of the coronary arteries (3 cases); small chronic inflammatory infiltrates of pericardium (2 cases)
Chang et al. [10]	2010	Mitogenic	Hypertrophy of myofibers; elongated, enlarged, and hyperchromatic nuclei with clumped chromatin; caterpillar nuclei; mitotic activity
Okamoto et al. [11]	1993	End-stage HCM	Massive fibrosis and marked disarray in residual hypertrophic myocardial fibers of the ventricular septum and both ventricular free walls; no coronary intramural lesions
Chen & Zhang. [12]	2006	dnr	Hypertrophy of myocardial fibers (all cases); increased interstitial connective tissue (all cases); interstitial myocardial fibrosis (4 cases)
Samanta et al. [13]	2011	Idiopathic right ventricular	Thinned out wall of the RV with individual myocardial fiber size variation, with marked anisonucleosis, interstitial edema, patchy interstitial, and perivascular fibrosis and endocardial sclerosis; no chronic or acute ischemia signs
Matoba et al. [14]	1990	Inflammatory DCM	Diffuse interstitial fibrosis in the subendocardial regions of the left ventricular wall; no massive replacement fibrosis nor inflammatory cell infiltrations
Simoes et al. [15]	1992	Idiopathic	Diffuse pattern of myocyte hypertrophy intermingled with myocytolysis and atrophied cardiac fibers; necrotic areas and mononuclear cell infiltrates in the myocardial infarction region; no evidence of myocardial fibrosis and no arteritis; scarring of the anteroapical region with interstitial edema and a focal perivascular mononuclear cell infiltrate
Takahashi et al. [16]	2008	dnr	Endocardial thickening with laminar deposition of elastic and collagen fibers in both ventricles; no intima and thickening in the atria; no fibrosis nor necrosis; scattering of mild to moderate interstitial lymphocytic infiltration in both ventricles and right atrium
Zhang et al. [17]	2013	dnr	dnr
Crauciuc et al. [18]	2021	Alcoholic	Amorphous sarcoplasm cells with an absent characteristic transversal striation; myocardial hypertrophy with thickened myofibrils; moderate interstitial fibrosis; lymphocytes in the myocardial interstitium
Ito et al. [19]	2021	dnr	Cardiomyocyte hypertrophy and elongation; nuclear pleomorphism; diffuse interstitial fibrosis; myofibrillar loss

**Table 3:** Genetic findings. DMD: dystrophin gene. RYR2: ryanodine receptor 2 gene. CTNNA3: catenin alpha 3 gene. DES: desmin gene. TNNT: troponin T gene. BAG3: Bcl2-associated athanogene 3. DSP: desmoplakin gene. PKP2: plakophilin-2 gene. TNNI3: troponin I3 gene. NAMPT: nicotinamide phosphoribosyltransferase gene. KCNE: potassium voltage-gated channel subfamily E. ACTC1: cardiac muscle alpha actin gene. KCNJ5: potassium channel subfamily member 5. TXNRD2: thioredoxin reductase 2. FLNC: filamin C gene. CASZ1: castoric zinc finger 1. TTN: titin gene. MYBPC3: myosin-binding protein C gene. MYH7: myosin heavy chain 7 gene. LMNA: lamin a/c gene. NKX2-5: nk2 homebox 5 gene. KCNQ1: potassium voltage-gated channel subfamily Q member 1. RBM20: RNA-binding motif protein 20 gene.

Authors	Year of Publication	Sample	Gross/Microscopic Data	Gene/Nucleotide or Amino Acid Change	Mutation Type
Afzal & Kristensen [20]	2008	8 DCMs	+	DMD (30% of cases)	dnr
Pelletti et al. [21]	2021	1 DCM	+	RYR2 -> c.4750C>A (p.Pro1584Thr) CTNNA3 -> c.1187T>G (p.Leu396Val)	Missense mutation
Callon et al. [22]	2024	1 DCM	+	DES -> c.1315G>A (p.Glu439Lys)	Missense mutation
Fernlund et al. [23]	2017	1 DCM	+	TNNT -> (c.518G>A (p.Arg173Gln) BAG3 -> (c.785C>T (p.Ala262Val)	Missense mutation
Ruppert et al. [24]	2004	45 DCMs; 62 controls	−	NADH dehydrogenase subunit genes: c.4079A>G p.Tyr1360Cys c.12347A>G p.His4116Arg c.12484G>C p.Pro4162Ala c.10385A>G p.Lys3462Asn c.10387G>C p.Gly3463Ala c.12412C>T p.Pro4138Ser c.12484G>C (repeated) p.Pro4162Ala c.12674A>G p.Asn4225Ser c.14965A>G p.Asn4989Ser c.14865G>A p.Cys4955Thr c.15068C>G p.Leu5023Val c.15172C>G p.Tyr5058His Cytochrome c oxidase subunit genes: c.5973G>A p.Ala1991Trp c.7042T>G p.Val2348Asp c.9484T>C p.Phe3162Thr c.9499T>G p.Phe3167Cys	Missense mutation
Elliot et al. [25]	2010	37 DCMs (23 familial)	−	Case 1: DSP-IVS15 1G C (abnormal splicing) PKP2-c.2630C A (H877Q) Case 2 and 3: PKP2-c.419C T (S140F) Case 4: PKP2-c.419C T (S140F) DSP-c.8134G A (A2712T) Case 5: DSP-c.2765_2766delCA (S922fsX928)	Missense mutation: PKP2-c.2630C A (H877Q); DSP-c.8134G A (A2712T)
Murakami et al. [26]	2010	18 DCMs	−	Case 19: TNNI3 -> Pro16Thr	Missense mutation
Dou et al. [27]	2015	394 DCMs, 395 controls	−	NAMPT -> (rs9034, rs2505568, rs61330082)	Single nucleotide polymorphisms
Mates et al. [28]	2018	136 DCMs	−	Cases 1–2: DSP -> (NG_008803.1) exons 21–23 Case 3: DMD -> (NG_012232.1) exons 45–62 Case 4: KCNE1 -> (NG_009091.1) exon 3d KCNE2-> (NG_008804.1) Case 5: ACTC1 -> (NG_007553.1) exons 2–7 Case 6: KCNJ5 -> (NG_023406.2) exons 2–3d	Deletion/Duplication
Rojnueangnit et al. [29]	2019	3 DCMs	−	Case 1: TXNRD2 -> c.1341T>G (p.Tyr447) Case 4: FLNC -> c.6291_6299dupAGT CAC CTA (p.Tyr2100)	Nonsense/Duplication
Orlova et al. [30]	2022	1 DCM	−	CASZ1 -> c.3781del p.(Trp1261GlyfsTer29)	Deletion
Hespe et al. [31]	2023	4 DCMs	+	Case 3: FLNC -> c.4926_4927insACGTCACA (p.Val1643Thrfs*26) Case 4: ndr Case 5: FLNC -> c.4926_4927insACGTCACA (p.Val1643Thrfs*26) Case 6: FLNC -> c.617G>A (p.Trp206Ter)	Insertion/Deletion/Nonsense
Dong et al. [32]	2024	3 DCMs	+	Case 1: TTN -> c.35485+1G>A Case 2: TTN -> c.82137del resulting in p.A27380Lfs*3 Case 3: TTN -> c.80415insA resulting in p.V26806Sfs*3	Splicing/Deletion/Insertion
Jin et al. [33]	2022	1 DCM	+	MYBPC3 -> NM_000256.3: C.24a>c, p.P8P	Synonymous variant
Kraoua et al. [34]	2024	3 DCMs	−	TNNI3 -> c.204delG; p.(Arg69AlafsTer8)	Deletion (frameshift)
Saxton et al. [35]	2024	79 DCMs	+	TTN -> 1. c.55104_55105delCA (p.Phe18368LeufsTer7) 2. c.89943_89959dupAAATAAAGTACCTGTGA (p.Thr29987LysfsTer6) 3–4. c.34935dupT(p.gln11646SerfsTer26) 5. c.66284G>A (p.Trp22095Ter) 6. c.59791C>T (p.Arg19931Ter) FLNC -> 1. c.531delC(p.lle178SerfsTer74) 2. c.1997delc (p.Asp399GlufsTer15) MYH7 -> c.2606G>A (p.Arg869His) DSP -> c.4751delC (p.Ala1584GlyfsTer18) TNNT2 -> c.452G>A(p.Arg151Gln) LMNA -> c.356+3_356+6delGAGT NKX2-5 -> c.435C>A (p.Phe145Leu) RYR2 -> c.14803G>A (p.Gly4935Arg) KCNQ1 -> c.085A>G (p.Lys362Arg)	Missense mutation/Deletion
Lorca et al. [36]	2025	5 DCMs	+	RBM20-> p.Arg636Cys	Missense mutation

## Data Availability

The original contributions presented in this study are included in the article/Supplementary Materials. Further inquiries can be directed to the corresponding authors.

## Supplementary Materials

The following supporting information can be downloaded at: https://www.mdpi.com/article/10.3390/diagnostics15233063/s1, Supplementary Material File S1: PRISMA 2020 Checklist.
